# Three-Dimensional Chipless RFID Tags: Fabrication through Additive Manufacturing

**DOI:** 10.3390/s20174740

**Published:** 2020-08-21

**Authors:** Sergio Terranova, Filippo Costa, Giuliano Manara, Simone Genovesi

**Affiliations:** Dipartimento di Ingegneria dell’Informazione, Università di Pisa, 56122 Pisa, Italy; sergio.terranova@ing.unipi.it (S.T.); filippo.costa@unipi.it (F.C.); giuliano.manara@unipi.it (G.M.)

**Keywords:** 3D printing, additive manufacturing, Radio Frequency IDentification (RFID), chipless RFID, mounted on metal

## Abstract

A new class of Radio Frequency IDentification (RFID) tags, namely the three-dimensional (3D)-printed chipless RFID one, is proposed, and their performance is assessed. These tags can be realized by low-cost materials, inexpensive manufacturing processes and can be mounted on metallic surfaces. The tag consists of a solid dielectric cylinder, which externally appears as homogeneous. However, the information is hidden in the inner structure of the object, where voids are created to encrypt information in the object. The proposed chipless tag represents a promising solution for anti-counterfeiting or security applications, since it avoids an unwanted eavesdropping during the reading process or information retrieval from a visual inspection that may affect other chipless systems. The adopted data-encoding algorithm does not rely on On–Off or amplitude schemes that are commonly adopted in the chipless RFID implementations but it is based on the maximization of available states or the maximization of non-overlapping regions of uncertainty. The performance of such class of chipless RFID tags are finally assessed by measurements on real prototypes.

## 1. Introduction

Additive Manufacturing (AM) is a rapid prototyping method of fabrication based on three-dimensional (3D) printing. Differently from the more traditional technologies based on a subtractive process (e.g., milling) or a formative one (e.g., forging), AM creates the desired shape starting from a three-dimensional Computer-Aided Design (CAD) model that is sliced into a set of layers, each one being a thin cross-section of the original model. The obtained object is a staggered approximation whose fidelity with the original one depends on the finite thickness of each layer, the properties of the employed material and the adopted AM technology [1]. The choice can be made among processes that exploit photopolymerization, such as the Stereolithography (SLA), material extrusion, as in the case of Fused Filament Fabrication (FFF), and power bed fusion, like Selective Laser Sintering (SLS), just to name a few that can print plastic-based materials, ceramics and even metal [2].

Two remarkable features provided by AM consist in providing the ability to build very complex geometries and in tailoring mechanical and electrical parameters of an object by using the capability of controlling the density of a printed object [3]. This unique combination has opened new and exciting paths in several areas that span from aerospace to medical, from transportation to energy [4]. Furthermore, there are numerous examples of 3D-printed devices suitable for chemical-, temperature- and pressure-sensing applications [5,6,7]. AM has also boosted new solutions in electromagnetics where it has been adopted for lenses [8], Frequency Selective Surfaces (FSS) [9], printed electronics [10], reflectarray [11], antennas for space [12,13] and several other devices [3,14,15,16,17]. Among the most interesting and profitable application areas, the Radio Frequency IDentification (RFID) is certainly one of the more appealing and fast-growing [18]. The most widespread identification device consists of an antenna connected to an Integrated Circuit (IC) that modulates the scattered field, commonly referred to as a RFID tag [19]. The interrogation process of the RFID tag differs depending on the frequency bandwidth employed, which, in turns, determines the read range. Low-Frequency (LF) tags work around 130 KHz and use near-field inductive coupling to obtain the necessary power to communicate their information to the nearby reader (typically 10–20 cm). High-frequency (HF) tags work at 13.56 MHz, exploit near-field coupling as well and achieve similar read range. The Ultra-High-Frequency (UHF) range is adopted by RFID tags that commonly resonate in a narrow band within the 860–960 MHz frequency interval. The interrogating signal is a circularly polarized plane wave, and this kind of tag guarantees a reliable communication range up to 10 m.

Recently, a class of RFID tags that do not rely on an IC for encoding the information, the chipless RFID tags, have been proposed for both identification and sensing duties [20,21,22]. The vast majority of chipless RFID designs assume a plane wave interrogation system that must provide the proper amount of energy to be scattered back by the tag and recollected to the reader into an intelligible form. The information is generally encoded in the Frequency Domain (FD) or in the Time Domain (TD), and particular attention has to be paid to isolate the meaningful electromagnetic field reflection of the tag from the undesired backscattering noise generated by clutter and antenna coupling [23,24,25].

The most common chipless tag operating in time domain is based on Surface Acoustic Waves (SAW), which encode the information exploiting the time of travel of surface waves to a set of suitably placed reflectors. However, this solution is not cheap if compared to UHF RFID tags and may require a not negligible footprint [26]. The most diffused FD-based chipless tags instead encode information into the spectrum using resonant structures. The encoding algorithm is based on a one-to-one association among an information bit and the presence or absence of a resonance peak in the spectral signature at a predetermined and predictable frequency. These tags are robust to interference, fully printable and have large data capacity, although they require a broad spectrum for data encoding and a wideband-dedicated RFID reader. Currently, there are also many hybrid approaches between FD and TD systems [27,28,29,30]. Recently, printed dielectric encoders based on linear chains of dielectric inclusions were presented in Reference [31], in which a line of equally spaced resonators was sequentially read by linearly moving them in front of a near-field reader.

The reading range of these solutions can be comparable with the UHF tag, although a large frequency bandwidth is generally required. In order to overcome this bandwidth requirement, new encoding algorithms have been presented in the literature, such as in Reference [32], where the information is encoded in the quantized values of the difference between the TE and TM phase response and a multifrequency reader can be adopted. Although it is possible to cope with the backscattering noise adopting various strategies [33,34,35,36], for some applications, it is not required to achieve read ranges greater than a few centimeters, and therefore, near-field reading schemes have been employed even for chipless RFIDs. This can be the case when sensitive information is transferred or for increased data capacity or monitoring purposes [37,38].

The degrees of freedom offered by AM offers the possibility to consider unconventional RFID tag designs that can be easily manufactured by using inexpensive materials and low-cost processes. In the proposed concept study, a cylindrical dielectric structure is manufactured with inner voids that are invisible from outside and with an exterior appearance of a solid bulk shape. The three-dimensional (3D) chipless tag is completely realized with cheap material, and it is easy to manufacture with low-cost 3D printers. The proposed design provides an interesting coding capacity and, at the same time, conceals the information in its inner structure that makes it suitable for envisioned physical-layer security tasks and also for anticounterfeiting purposes [39,40]. The 3D chipless RFID tag can be mounted on metal platforms, and it is easily scalable to other dimensions or frequency bands. The presented 3D chipless tag represents an example of a frequency-domain chipless encoder that fully takes advantage of the three-dimensional nature of the tag and, at the same time, conceals the encoded information. The manuscript is organized as follow. Section 2 describes the geometry of the tag and its working principle. Section 3 describes the encoding and decoding scheme. In Section 4, the tag performance is experimentally verified. Conclusions are drawn in Section 5.

## 2. 3D-Printed Chipless RFID Tag

The adoption of an additive manufacturing process for realizing a device capable of storing information has been stimulated by the high flexibility offered by 3D with respect to 2D printing. The idea at the basis of the 3D chipless RFID design is to choose a tag shape that can exhibit a multi-resonance response in the frequency domain that, in turn, is exploited to encode the information. The reading system should be able to read this frequency signature once placed in contact with the tag. Polylactic acid (PLA) has been chosen for the tag fabrication due to its low-cost and easiness of use, even with entry-level printers.

The theoretical framework of the idea at the basis of the investigated 3D chipless tag comes from the considerable existing literature on dielectric resonators [41] and Dielectric Resonator (DR) antennas [42,43,44,45], and, in particular, on dual frequency and wideband DR antennas [46,47,48]. Dielectric resonators are microwave devices with high-quality factor *Q,* and they are used as elements in microwave filter and oscillator designs since these are excellent substitutes for metal resonant cavities. In a DR, it is possible to store electromagnetic energy because, once a field is triggered inside the resonator, an electromagnetic wave propagates and bounces back and forth between the walls, giving rise to a standing wave. Generally, this type of behavior is obtained when the dielectric permittivity is very high (e.g., 40–50). This effect can be well explained by calculating the reflection coefficient at the dielectric–air interface.

(1) $$\Gamma =\frac{\sqrt{\varepsilon_{r}}-1}{\sqrt{\varepsilon_{r}}+1}$$

As can be seen in Equation (1), the coefficient becomes equal to 1 when the dielectric constant becomes large. It is, therefore, possible to further approximate the air–dielectric interface according to the non-physical condition of Perfect Magnetic Conductor (PMC), which requires the tangential components of the magnetic field to vanish. Although the assumption of the PMC condition has been profitably exploited for the design of dielectric resonators, it has always been known that a portion of the electromagnetic field can leak from the resonator, leading to a decrease of the *Q*-factor. This important observation led to the first studies of the irradiation of a DR in Reference [49], and since then, several papers have been published on this subject. In order for the dielectric material to operate as an antenna, its permittivity must be moderate, approximately in the 5–30 range, so that the energy in the cavity can escape from the walls and be irradiated into the environment. It is therefore important to underline that the proposed 3D tag design can benefit from the dielectric resonator antenna theory, but in many ways, it differs because the theoretical models present in literature are valid only under the condition of high permittivity, while in the present case, the PLA printing filament is characterized by a low permittivity of 2.62.

**Figure 1:**
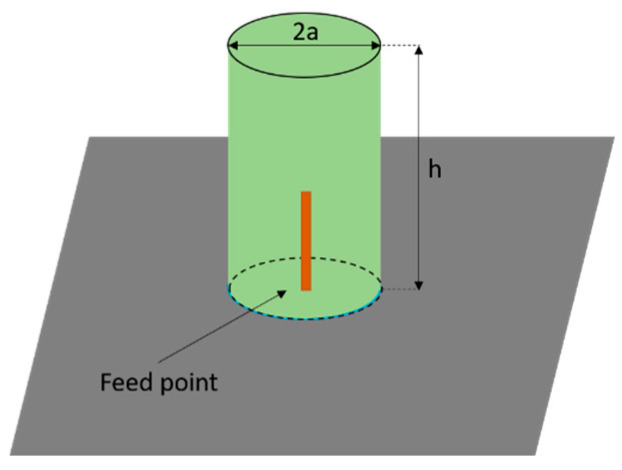
Three-dimensional (3D) view of the probe-fed cylindrical DRA.

In order to explain how these theoretical principles can be exploited for designing a 3D chipless tag, it is useful to briefly summarize the behavior of a cylindrical Dielectric Resonator Antenna (DRA) (Figure 1).

Depending on the position of the probe, one of the principal radiating modes can beexcited [41,44,45]. The length of the coaxial probe, on the other hand, is sized to handle impedance matching. For a probe positioned in the center of the dielectric cylinder, as in our case, $TM_{01\delta }\;$mode is excited. In addition, thanks to this type of coupling, a matching network is not required. Clearly, this length must be less than the height of the dielectric cylinder to avoid the probe radiation. It is interesting to notice that fundamental $TM_{01\delta }\;$mode radiates like a short electric monopole. It should also be specified that this resonant mode coincides with that of the cylindrical DRA only in the semi-space z > 0. The distribution is in fact obtained by eliminating the ground plane and applying the method of images theory.

Unfortunately, there are no simple relationships to dimension the probe height once the dielectric cylinder dimensions are fixed. However, through an intensive campaign of experimental investigations and curve fitting, useful relationships have been obtained to calculate the resonance frequency and the merit factor associated with a particular resonant mode [45]. The approximated expression used to calculate the resonance frequency of the $TM_{01\delta }\;$is reported in Equation (2), which is also suitable for low permittivity values.

(2) $$f_{R}=\frac{c}{2\pi a\sqrt{\varepsilon_{r}+2}}\sqrt{3.83^{2}+{\left(\frac{\pi }{2}\right)}^{2}{\left(\frac{a}{h}\right)}^{2}}\;\;\;\;\;\;\;\;\;\;\;\;\;0.125\leq \frac{a}{h}\leq 5$$

**Figure 2:**
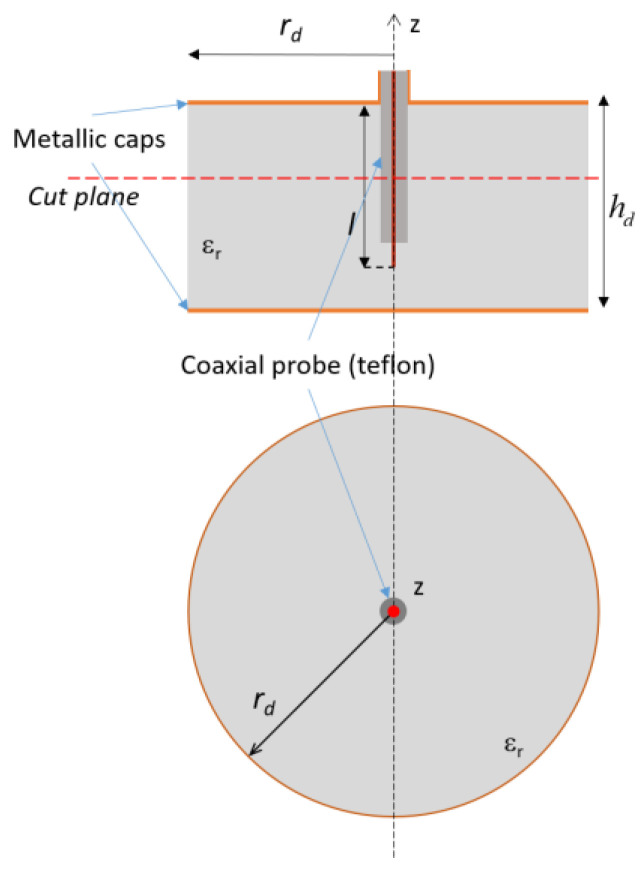
Lateral and top view of the first iteration for the tag design process.

**Figure 3:**
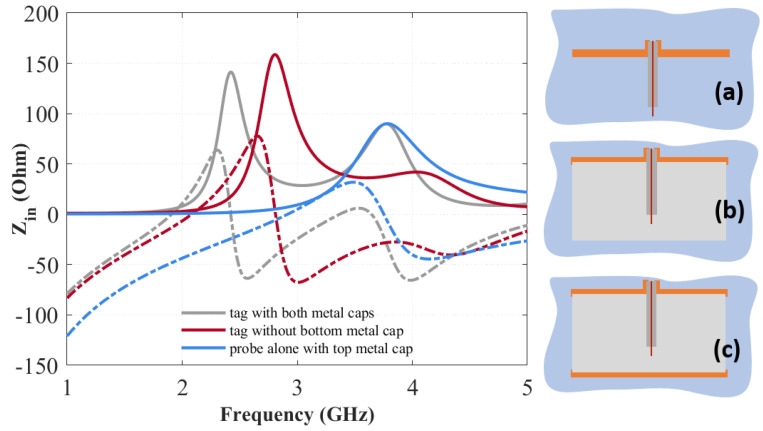
Real (continuous) and imaginary (dashed) part of the input impedance as a function of frequency considering the probe alone placed on the top metal cap (**a**), the dielectric structure without the metal bottom cap (**b**) and with both metal caps (**c**).

The first iteration of the 3D tag design process is shown in Figure 2, where a structure consisting of a solid cylinder of PLA (ε_r_ = 2.62 − j0.05), with metallized top and bottom face, is considered. This tag is probed by using a standard coaxial connector introduced at the tag center. The dimensions of the tag (*h_d_*, *r_d_*) are set by considering the cutoff frequency of the fundamental TM_01δ_ mode of the dielectric structure. Since the predefined frequency bandwidth has been set within 1.0 and 5.0 GHz, the radius, *r_d_*, is set equal to 35 mm. The coaxial probe (*l* = 18 mm) is employed as a tag reader, and the collected measured impedance provides the input data for the data-encoding process. The effects of the coupling between the probe and the tag are described in Figure 3, where the real and imaginary part of the tag input impedance (*Z_in_*) are reported. The case of the probe placed on the top metal cap and radiating in free space is obviously similar to that of a monopole with a finite ground plane. There is a first resonance around 3 GHz in correspondence of a low value of the real part of the impedance and then an anti-resonance around 3.8 GHz, with a higher real component. The loading effect of placing the 3D structure dielectric structure around the probe determines the downshift around 2 GHz of the resonance and 2.8 GHz of the antiresonance, and an increase of the real part. A hint of another couple, resonance/antiresonance, starts to be visible within the 3.5–4 GHz band.

In order to explain the chosen geometric parameters of the 3D tag, the above-mentioned expression (2) can be adopted. By using the values of height, *h_d_ =* 22 mm, radius, *a* = 35 mm and ε_r_ = 2.62, a resonance frequency equal to *f_R_* = 2.902 GHz is obtained. This resonance frequency deviates by about 100 MHz from the simulated resonance, as can be seen from the red curve in Figure 3B, where the imaginary part of the impedance crosses the zero level at *f_R_* = 2.801 GHz. Once this first project was done, it was thought to add a metal cap in order to avoid an antenna behavior and make this tag similar to a dielectric resonator, despite its low permittivity ε_r_.

**Figure 4:**
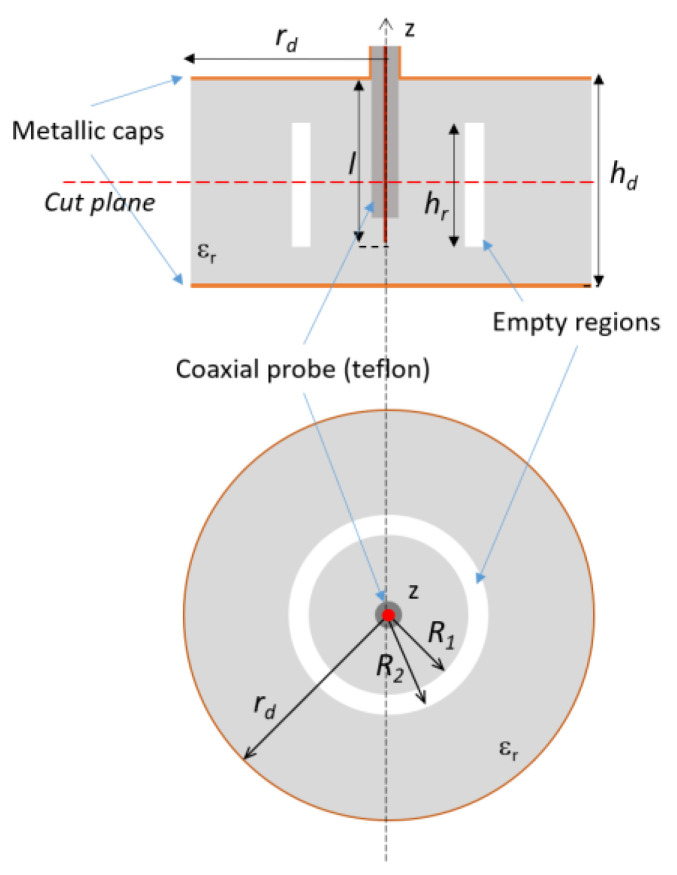
Lateral and top view of the second iteration of the design process in which an empty annular sector is introduced.

A second metallic cap is then added to the bottom surface of the structure to provide an isolation from objects on which the tag could be placed, even metallic ones. This tag exhibits a resonance/antiresonance at lower frequency but also a more pronounced sign of another one around 3.5 GHz. It could be useful for encoding purposes to fully excite this latter one as well in order to possibly exploit it for encoding purposes. Therefore, the next step is to find a way for enhancing the resonance/antiresonance couples and be able to vary their position. To this aim, in view of the degrees of freedom offered by the additive manufacturing process, an empty annular sector has been included inside the original dielectric structure (Figure 4).

**Figure 5:**
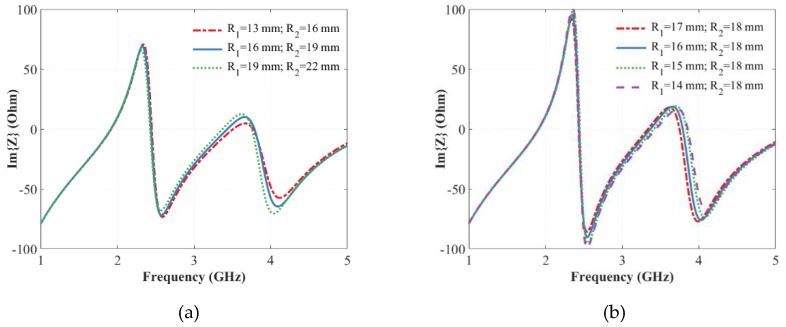
Imaginary part of the input impedance as a function of frequency in case the width and the height of the empty ring are fixed (**a**), and by varying the width of the empty ring for a fixed R_2_ (**b**).

The effects of changing the dimensions of this ring-shaped void are reported in Figure 5, for the case of a fixed thickness (i.e., *R_2_*-*R_1_* = constant) and for a variable thickness when *R_2_* is assigned. As it can be seen from Figure 5a, by fixing the thickness and the height of the empty ring-shaped void to 3 and 14.5 mm respectively, the position of the second couple of resonance/antiresonance can be changed by varying *R_1_* and *R_2_*. The resonance shift is also obtained by varying the thickness of the vacuum inclusion and fixing one of the two radii, for example *R_2_* (Figure 5b). These frequency shifts of the second resonance or antiresonance can be exploited for encoding purposes, although other variable zero-crossings of the imaginary part of the impedance are needed for increasing the number of codified states.

**Figure 6:**
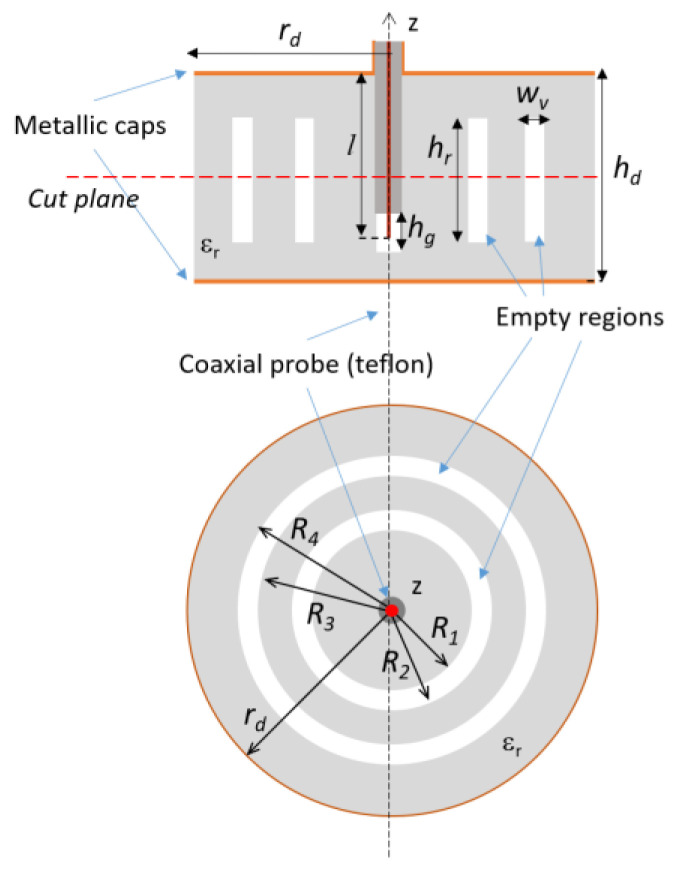
Lateral and top view of the third iteration of the design process in which an empty annular sector is introduced.

The previous analysis has suggested that a void inclusion is helpful in shifting the resonances. Therefore, an additional void inclusion is added (Figure 6).

**Figure 7:**
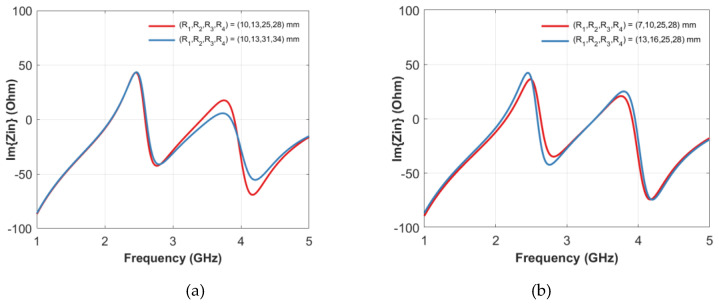
Imaginary part of the input impedance as a function of frequency in case the first empty ring is fixed and the position of the second one is changed (**a**), and by fixing the second empty ring and changing the position of the first one (**b**).

An exhaustive study of the possible configurations of the structure presented was carried out using Ansys High-frequency structure simulator (HFSS) [50] to investigate the behavior of the two pairs of resonances as a function of the void inclusion radius and reciprocal position. In more detail, with *R_1_* and *R_2_* being the inner and outer radius of the first inclusion and *R_3_* and *R_4_* those of the second one, a set of configurations has been considered in which the dimension and position of the first inclusion are fixed (i.e., *R_1_* and *R_2_* are constant) while the position of the second ring is changed (i.e., *R_3_* and *R_4_* are variable) and only the thickness is kept constant (i.e., *R_4_* and *R_3_* are constant). It is apparent that the first resonance is not affected by this change, whereas the first antiresonance and, more significantly, the second couple, resonance/antiresonance, are shifted (Figure 7a). On the contrary, fixing the dimensions of the second ring (*R_3_*, *R_4_*) and varying the position of the first ring does not affect the second resonance but alters all the other zero-crossing of the impedance imaginary part (Figure 7b). It can be concluded that the resonance/antiresonance related to each void inclusion cannot be controlled independently and that a change in one inclusion alters the whole frequency response. It is, therefore, necessary to map the resonance/antiresonance couples of all the allowed configurations to introduce a suitable coding of the electromagnetic signature. In particular, the height of the rings is set equal to *h_r_* = 14.5 mm and the width of the voids. *w_v_* = 3 mm (Figure 7). By considering the dimension of the tag and the manufacturing process, it has been considered to vary *R_1_* within (4–15) mm, *R_2_* in (7–18) mm or up to half of the radius of the dielectric cylinder and *R_3_* within (19–31) mm and *R_4_* in (22–34) mm, or at 1 mm from the external surface of the cylinder.

**Figure 8:**
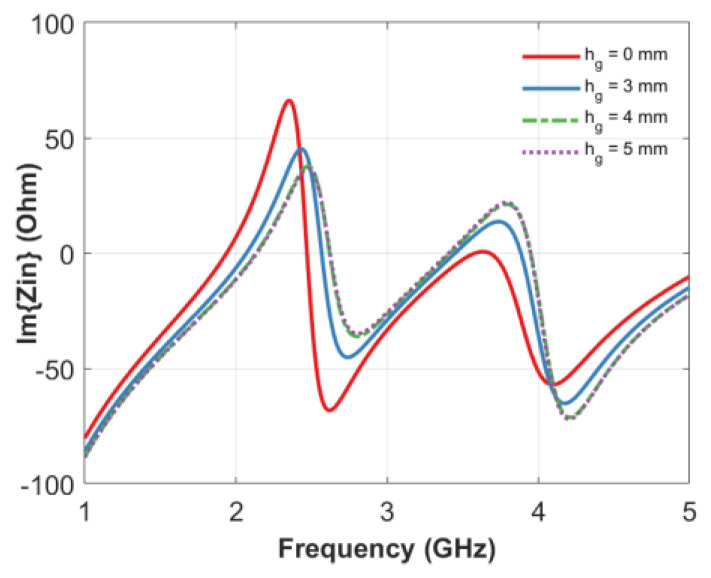
Effect of the presence of an air gap around the connector tip and the dielectric material (see Figure 6) on the imaginary part of impedance Im(z). All curves refer to a tag with total height h_d_ = 22 mm, height of void inclusions h_r_ = 14.5 mm and void inclusions radii R_1_, R_2_, R_3_, R_4_ = 10, 13, 25, 28 mm, respectively.

In view of the maximization of the number of the states, it is important that the imaginary part of the impedance has a large dynamic so as to have unambiguous and non-intersecting areas associated to the encoding based on resonance frequencies. To achieve this goal, an air gap, *h_g_*, has been introduced in the design (Figure 8).

In fact, by varying this latter parameter from 3 to 5 mm, it is possible to observe a significant shift of the resonance frequencies (Figure 8) and therefore, an increase of the potential number of states. It is interesting to note that in the absence of an air gap, the second resonance around 3.6 GHz is hardly detectable. By increasing the value of h_g_, the zero-cross is much more easily detectable. When the height of the air gap is 3 mm, it reaches the tip of the probe of length *l*. By further increasing the value of h_g_ from 4 to 5 mm, the tip of the probe is no longer in contact with the dielectric material, and it can be seen that then, the curves are practically superimposed. Looking at the reported trend, a value of h_g_ = 4 mm has been chosen since a further increment does not provide significant impedance variations.

## 3. Encoding and Decoding Scheme

Most of the adopted encoding algorithms for chipless RFID tags are based on ON/OFF schemes applied to the amplitude of resonance peaks in the electromagnetic response of the device. This approach is also encouraged by the fact that each peak is associated with a resonator, sometimes referred to as a resonating particle, that can be activated (or removed) in order to apply the coding. Since the proposed resonator does not rely on any single independent particle but on the overall interactions among the voids inside the dielectric structure, it has been of fundamental relevance to conceive a coding strategy that manages to extract useful information from the spectral signatures of a 3D chipless tag. This task has been accomplished by exploiting the correlations that exist between the variations of the geometric parameters of the tag, such as the positions and dimensions of the voids, that have a non-predictable effect on the resonances.

The algorithm is based on a positional resonance encoding, so each pair of resonances that can be embedded in the spectral signature can be represented as a point on a two-dimensional (2D) map, whose axes identify the possible values that each resonance can assume. The main purpose of the algorithm is then to unambiguously distinguish two points on this map that represent the coded information, considering, at the same time, that they can slightly change because of imperfections and errors due to the reading or manufacturing. It is possible to model this range of indecision as a certain area (coding zone) centered around the ideal coded position. So, the algorithm aims to find the largest non-intersecting subset of coding zones in the plane. Usually, the problem is not easy to solve because it belongs to the class of Non-determistic Polynomial-time hard problems (NP-hard problems). Algorithms of this type take the name of MISR (Maximum Independent Set of Rectangles) and can be solved, although in an approximate way when the considered coding zone has the shape of a rectangle.

**Figure 9:**
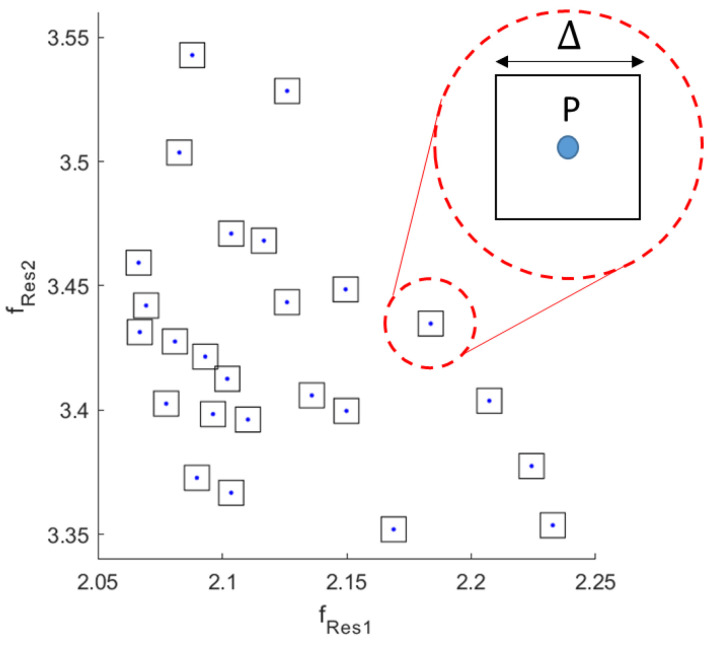
Representation of the tolerated uncertainty regions obtained by considering the first and Table 3. mm, tag height is 22 mm and the height of the rings is 14.5 mm.

At each allowed configuration of the resonator, that is a quadruple (*R_1_*, *R_2_*, *R_3_*, *R_4_*), a state is associated, which is defined by using the first two resonance frequencies (*f_Res_*_1_, *f_Res2_*). In order to define an unambiguous encoding scheme, each state is mapped into a point *P* of coordinate (*f_Res1_*, *f_Res2_*) in the two-dimensional space defined by the resonance frequencies exhibited by the tag, under the assumed geometrical constraints. In order to elaborate a robust encoding scheme, it is also considered that measurements on the real device may differ from the expected ones due to, for example, small imperfections in the realization of the prototype or the deterioration of the employed material. For this reason, a certain percentage tolerance in the mapping of each couple of frequencies into a codified state has been considered. This implies that the state is not codified by a single couple of resonances (i.e., point *P*), but by an area around the point *P* representing the state. For the sake of simplicity, in the hypothesis of an error Δ, it may consist of a certain percentual change of each resonance frequency or of a fixed quantity not directly related to the resonant frequencies. In the former case, the error can be associated to a surface which has the form of a rectangle, whereas in the latter case, it is represented by a square. An example of this mapping is reported in Figure 9 by square regions (Δ = 10 MHz). This choice of considering a certain error clearly has an impact on the encoding phase, where it will be necessary to adopt a criterium for maximizing the states that can actually be distinguished, that means selecting the maximum number of regions that do not intersect.

**Figure 10:**
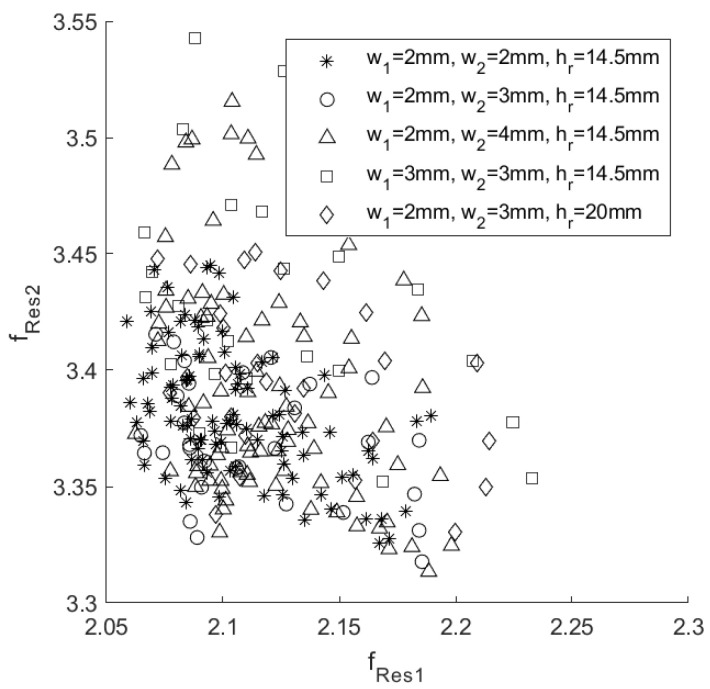
Representation of the couples of resonance frequencies of different tag families characterized by different thicknesses and heights. w_1_ is the thickness of the first empty ring, w_2_ is the thickness of the second one, and h_r_ is the height of both rings.

**Figure 11:**
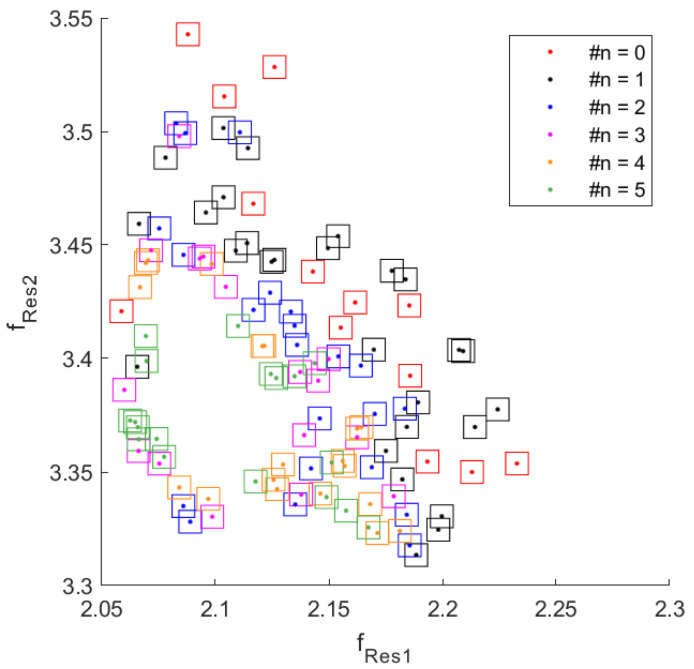
Regions of uncertainty classified according to the number of their intersections marked by the symbol # in the figure.

**Figure 12:**
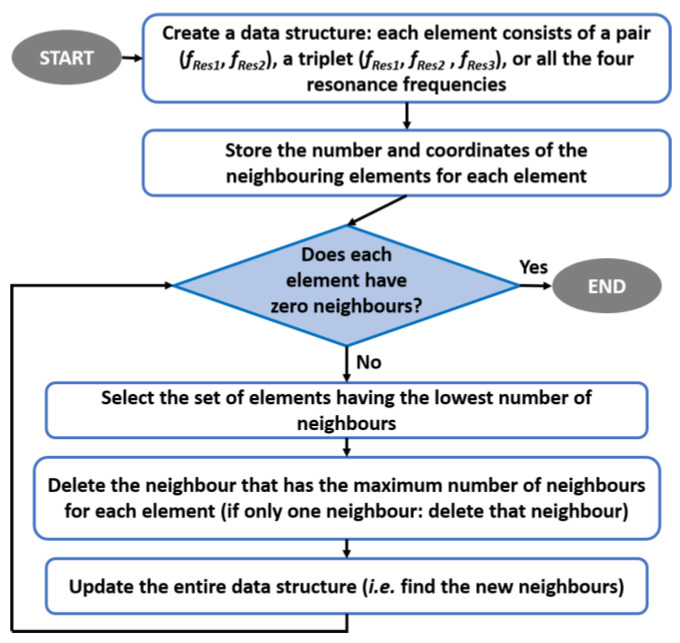
Flow chart of the algorithm to select the maximum number of unambiguous identification states.

It is, therefore, possible, in principle, to apply the encoding algorithm to a state comprising any pair of these zeros. Given a family of Im{*Z_in_*}, there are 6 possible pairs of frequencies (*f_i_*,*f_j_*, with *i*,*j* = 1, 2, 3, 4, *i* ≠ *j*) that can be considered. Once all the possible combinations have been tested, it will be possible to know which is the best pair of zeros of Im{*Z_in_*} that will provide the maximum number of distinguishable states suitable for decoding. In order to maximize the final number of states, a further set of device configurations has been considered, in which, other than the position of the empty annular regions, their thickness and height are varied. All the consequent resonance pairs (*f_Res1_*, *f_Res2_*) are shown in Figure 10. In particular, the configurations having different thickness and height are highlighted with a different marker. The initial step of the encoding algorithm is to create a data structure that stores the number and coordinates of the neighboring points for each resonance frequency pair (*f_Res1_*, *f_Res2_*), together with the information on those neighbors that intersect the area of the considered pair. In this way, it is possible to build an organized map of all disjointed points, highlighting points that have a number of neighbors equal to 1, 2, 3 and so on. In Figure 11, these rectangular regions are shown with different colors, depending on the number of neighbor points. For simplicity, only resonance frequency pairs that have up to 5 neighbors are represented, although the maximum order of intersections can be much higher. Next, the indices of the states that have the same number of neighbors are stored in separate vectors in order to consider sub-sets of the more general initial structure. The points that are classified as states with 0 neighbors are clearly disjointed and unambiguously identifiable, and they are therefore considered as resolved values. After this first selecting step, the remaining states are ranked in ascending order depending on the number of their neighbors. The group of configurations with only one neighbor (order 1) are the one considered at this stage. To avoid any possible ambiguity, the algorithm removes the neighbor state from those allowed and updates the entire data structure so that this deleted pair (which may have more than one neighbor) is also deleted from the list of other states. By having eliminated the only nearby node, the states that were ranked first now have no neighbors and so they are aggregated to the set of disjointed points. Once all nodes of order 1 have been resolved, the next order set, e.g., order 2, is considered. In this case, the list of states with 2 neighbors is scrolled, and for each of these, only one of the two neighbors is eliminated. In particular, the neighbor that has the maximum number of neighbors is deleted and also, this time, the whole data structure is updated and nodes of order 1 will be revealed. The above-stated criterion of eliminating the neighbor that has the largest number of neighbors is also valid if the order of the set is greater than 2. Applying this procedure iteratively always to the set having the lowest number of neighbors will finally lead to only states (regions) disjointed in frequency and therefore, distinguishable during the decoding phase. A comprehensive flow chart of this process is illustrated in Figure 12.

It is interesting to notice that the initial choice of the Im{Z_in_} zeros can lead to a different number of disjointed final states, as illustrated in Table 1. It is shown that by setting = 10 MHz, the best result is obtained when the third and fourth zero are chosen, which are the second resonance and second antiresonance, respectively. In this case, there are 105 distinguishable final states which correspond to 6.71 information bits. At the end of the encoding phase, all the possible separate regions associated with the couple of frequencies (*f_Res1_*, *f_Res2_*) have been selected. During the decoding phase, a search is performed through all the couples and the corresponding codified state is associated to the spectral signature. In more detail, it is checked if the couple of frequencies (f_1*_, f_2*_) related to the considered tag belongs to the intersection of the interval f_Res1_−Δ/2 ≤ f_1*_ ≤ f_Res1_ + *Δ/2* and f_Res2_−Δ/2 ≤ f_2*_ ≤ f_Res2_+Δ/2. If the condition is never verified, then the error made during the reading exceeds the estimated one and no decoding is possible.

## 4. Prototype Realization and Measurements

In order to evaluate the performance of the proposed detection system, several prototypes of the 3D chipless RFID tag have been fabricated by using a 3D PowerWasp EVO printer [51]. A vector network analyzer (Keysight E5071C, Keysight, Santa Rosa, California, U.S.) has been used to measure the spectral signature of the tag in a non-anechoic environment.

The g-code file for the printing process has been generated by using the Ultimaker Cura software [52]. The diameter of the adopted PLA filament is 1.8 mm, and the selected extruder temperature is 185°. The print bed was not heated, so it was necessary to spread glue on it to stabilize the tag during the filament deposition process. To further improve adhesion during printing, a filament mesh was created before printing the piece. This substrate has a thickness of 0.3 mm, and it is printed by stacking 3 layers, each 0.1 mm high. The printing speed adopted was 70 mm/s in order to guarantee a good tradeoff between a good surface finish and not too long manufacturing times. The height of the inner layers, which determines the vertical resolution, has been set to 0.1 mm, a sufficient value to guarantee a good resolution given the cylindrical symmetry of the object. These layers have been printed with an infill percentage of 80%, in order to achieve a good mechanical stiffness. Moreover, a sufficiently high prototype density is important to ensure the desired dielectric properties. Among the different printing patterns available (e.g., rectilinear, honeycomb, grid), the one used for the internal layers was the grid type. For the external layers, i.e., top and bottom surfaces, a rectilinear pattern and an infill percentage of 100% has been used. The adopted line width was 0.4 mm, which is equal to the nozzle size. The wall thickness determines the width of the vertical walls of the tag, and it must be a multiple of the nozzle size. However, increasing the thickness of the shell determines a significant increase in the printing time and, at the same time, a greater mechanical stiffness. Generally, a value of 0.8 mm is a good design choice, and this was the set value. Once the PLA part of the device has been printed, a 0.5 mm thick copper metal disc was applied to the top and bottom layer using a thin coat of glue.

**Figure 13:**
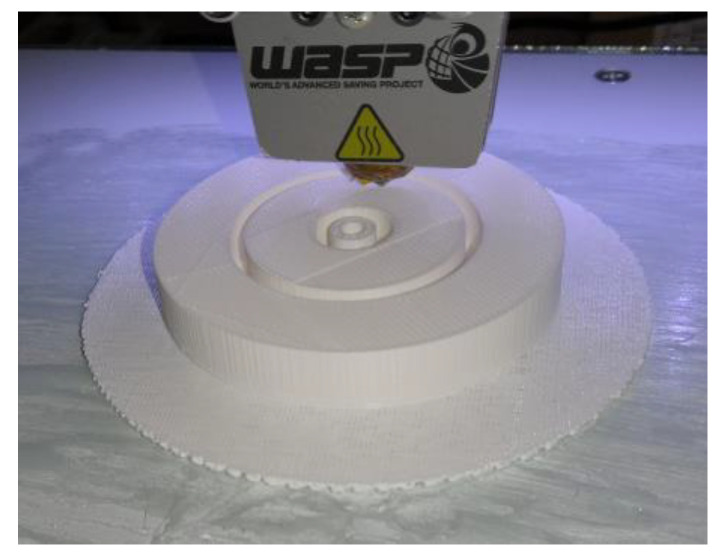
Three-dimensional (3D)-printed tag prototype during the realization phase.

**Figure 14:**
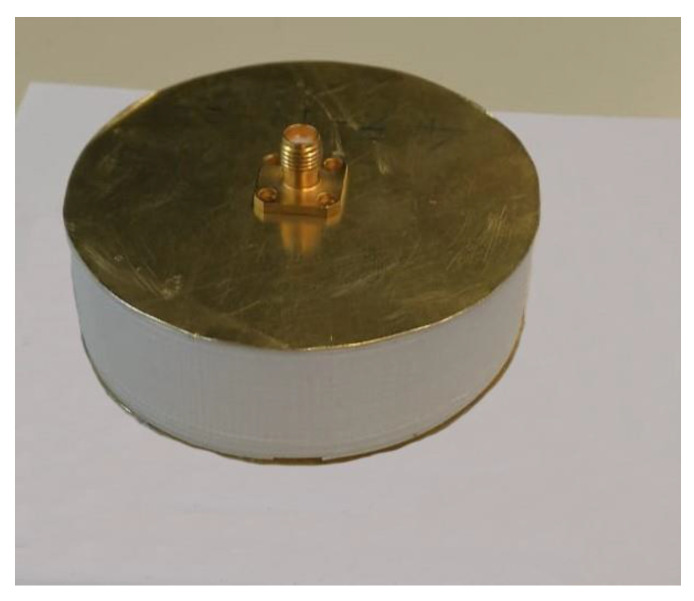
Final assembled prototype of the realized 3D chipless tag.

The 3D tag is shown in Figure 13, during the realization phase. It is worth noticing the presence of the two empty annular regions and the central hole in which the SMA connector will be inserted. It is also important to highlight that the additive manufacturing process allows a simple and seamless implementation of this prototype, whereas traditional techniques (i.e., subtractive ones) would have required the fabrication of the bulk cylinder, the drilling of the voids and a subsequent gluing to cover the inner structure. As a result, from the outside, it is not possible to infer the dimension of the inner voids (Figure 14).

**Figure 15:**
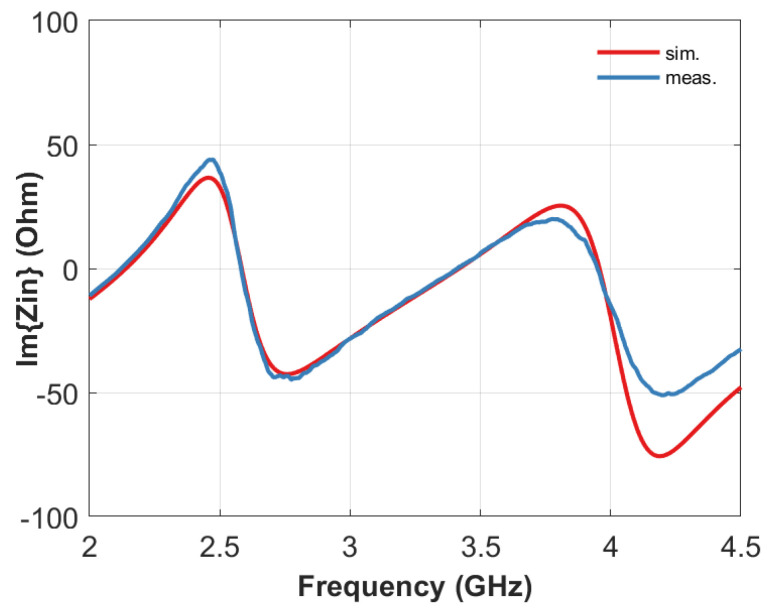
Comparison between simulated and measured results for the manufactured prototype (*h_d_* = 22 mm, *h_r_* =14.5 mm, *R_1_*, *R_2_*, *R_3_*, *R_4_* = 4, 7, 19, 22 mm respectively, *r_d_* = 35 mm and *h_g_* = 4 mm).

A representative measure of the input impedance exhibited by one of the tags is compared with the corresponding simulation result in Figure 15. The geometric parameters of the manufactured prototype are: *h_d_* = 22 mm, *h_r_* = 14.5 mm, *R_1_*, *R_2_*, *R_3_*, *R_4_* = 4, 7, 19, 22 mm respectively, *r_d_* = 35 mm and *h_g_* = 4 mm. The agreement is quite good for all the four resonance frequencies.

Different encoding sequences can be used depending on the number of considered frequencies (i.e., 2, 3 or 4) and the selected ones (i.e., *f_RES1_*, *f_RES2_*, *f_RES3_*, *f_RES4_*). Table 2 shows the range of variations associated with each resonance frequency. The number of available bits (*#bit*) has been calculated for all the possible options that can be exploited by using different combinations of the resonance frequencies for several values of error expressed in MHz, Δ (Table 3).

On the basis of the manufactured prototypes, it has been found that a value of Δ = 20 MHz can provide a reliable measurement and therefore an intelligible and unambiguous recovery of the encoded data. This means that in case that all four resonance frequencies are exploited, the proposed 3D chipless RFID can offer 6.6 bits.

Since the number of encoded states is strictly related to the number of resonances, it is apparent that increasing their number can enhance the number of bits. This can be obtained, for example, by using a material with higher dielectric permittivity or by exploring the possibility offered by multi-material 3D tags.

It is interesting to note that the presented structure is not based on a unique and irreproducible signature, such as chaosmetry-based methods. The aim is to maximize the information regardless of the correlation that exists between the resonance/antiresonance pairs of the tag. In this case, the superposition principle of effects cannot be applied, as it would happen for a tag made up of several resonators in which a sharp and predictable resonance occurs due to the presence/absence of a specific resonator, such as in the most common chipless RFID tag implementations. It is interesting to note that there are different geometric configurations (thickness of annular voids, radial distance of voids from the axis of the cylindrical structure, height of voids, etc.) that give rise to the same zeros of the resonances, although the entire signatures are different. It is, therefore, possible to exploit this simple fact in order to introduce a degree of unpredictability for anti-counterfeiting purposes, since only those who know the encoding scheme know which bit pattern corresponds to a particular vector of zeros, extracted from the imaginary part of the input impedance.

## 5. Conclusions

A novel Chipless 3D-printed tag, fabricated by using PLA filament and a 3D low-cost printer, has been proposed in this paper. The tag has been metallized on the top and bottom surface in order to obtain a resonant-type structure by confining the electromagnetic fields within it. In addition, thanks to the metallization, the tag can be easily mounted on metal surfaces. The reading process is performed by inserting a probe inside a small hole on the bottom surface of the tag. The proposed reading scheme is particularly recommended for anti-counterfeiting applications for avoiding unwanted leakage of information and ensuring a closed reading system. The information has been encoded in the imaginary part of the tag impedance and measured through a vector network analyzer. The employed design ensures good coding capacity. The fabricated tags appear identical through a visual inspection, with an external appearance of a solid cylinder, as the geometry of the specific tag configuration depends on the internal features. A data-encoding algorithm based on the maximization of available states or the maximization of non-overlapping regions of uncertainty has been presented. The design presented in this work can be extended to other geometric shapes as well as other alterations within the tag during the printing process. It is also possible to adopt other printing filaments, characterized by different dielectric properties, to adequately modify the electromagnetic response of the tag and further increase the coding capacity. An improvement in coding density can be achieved by adopting a high permittivity filament and including appropriate modifications, such as voids, inclusions with dielectrics of different permittivity or doped with metal powders in order to mimic a metal inclusion. In this way, the spectral signature of the tag, e.g., the imaginary part of the impedance or the phase of the reflection coefficient, may contain dozens of resonances and the latter ones can be exploited to greatly increase the storable information [53,54]. It is interesting to notice that by adopting a higher permittivity printing filament (e.g., $\varepsilon_{r}$= 10 or more [53]), the size of the tag and its weight can drop significantly and, although the cost of the material increases, the final cost of the tag remains almost unaffected.

## Figures and Tables

**Table 1:** Number (#) of encoded states and corresponding number of bits obtained by setting Δ = 10 MHz.

f_Res1_	f_Res2_	# of Values	# of Bits
1	2	49	5.61
1	3	84	6.39
1	4	102	6.67
2	3	73	6.19
2	4	90	6.49
3	4	105	6.71

**Table 2:** Range of values for each resonance frequency (MHz).

	Lower Value	Upper Value
***f_RES1_***	2058	2232
***f_RES2_***	2545	2660
***f_RES3_***	3313	3542
***f_RES4_***	3811	4060

**Table 3:** Number of available encoded states and bits for different combination of resonance frequencies and values of Δ, the error expressed in MHz.

f_RES1_	f_RES2_	f_RES3_	f_RES4_	#enc Δ = 10	#bit	#enc Δ = 20	#bit	#enc Δ = 30	#bit	#enc Δ = 40	#bit
1	2			49	5,6	17	4,1	10	3,3	7	2,8
1	3			84	6,4	37	5,2	22	4,5	15	3,9
1	4			102	6,7	45	5,5	25	4,6	17	4,1
2	3			73	6,2	32	5,0	17	4,1	12	3,6
2	4			90	6,5	37	5,2	20	4,3	13	3,7
3	4			105	6,7	45	5,5	28	4,8	19	4,2
1	2	3		117	6,9	44	5,5	24	4,6	14	3,8
1	2	4		145	7,2	61	5,9	29	4,9	18	4,2
1	3	4		190	7,6	89	6,5	51	5,7	32	5,0
2	3	4		177	7,5	80	6,3	44	5,5	27	4,8
1	2	3	4	197	7,6	96	6,6	52	5,7	33	5,0
